# Characteristics of [^18^F]FET PET and MRI in isocitrate dehydrogenase *(IDH)-*mutant gliomas diagnosed according to the WHO 2021 classification – a retrospective analysis

**DOI:** 10.1007/s00259-026-07760-2

**Published:** 2026-01-23

**Authors:** Enio Barci, Maximilian J. Mair, Jonas Reis, Katharina Müller, Jera Isakaj, Ergi Istrefi, Isabelle von Polenz, Sophie C. Siegmund, Lena Kaiser, Matthias Preusser, Christian Schichor, Niklas Thon, Patrick Harter, Louisa von Baumgarten, Robert Forbrig, Nathalie L. Albert

**Affiliations:** 1 Department of Nuclear Medicine, LMU University Hospital, LMU Munich, Munich, Germany; 2 Division of Oncology, Department of Medicine I, Medical University of Vienna, Vienna, Austria; 3 Institute of Neuroradiology, LMU University Hospital, LMU Munich, Munich, Germany; 4 Department of Neurology, LMU University Hospital, LMU Munich, Munich, Germany; 5 Bayerisches Zentrum für Krebsforschung (BZKF), partner site Munich, Munich, Germany; 6 Department of Neurosurgery, LMU University Hospital, LMU Munich, Munich, Germany; 7 German Cancer Consortium (DKTK), Partner Site Munich, Munich, Germany; 8 Neurosurgery Department, University Hospital of the Ruhr-University of Bochum, Bochum, Germany; 9 Center for Neuropathology and Prion Research, Faculty of Medicine, LMU Munich, Munich, Germany

**Keywords:** Glioma, Positron emission tomography, Magnetic resonance imaging, Amino acid PET

## Abstract

**Purpose:**

Amino acid positron emission tomography (PET) is increasingly utilized in the clinical workup of patients with glioma. To better understand the PET characteristics of *IDH*-mutant glioma according to the 2021 WHO classification of CNS tumours, this retrospective study explored the association between amino acid PET and magnetic resonance imaging (MRI) characteristics in patients with newly diagnosed *IDH*-mutant glioma.

**Methods:**

Patients with histologically verified *IDH*-mutant glioma who underwent [^18^F]FET PET/CT scans without prior treatment were included. PET parameters (PET-positivity according to PET RANO 1.0 criteria, maximal and mean tumour-to-background ratio (TBR_max_, TBR_mean_), PET volume and uptake kinetics including minimal time-to-peak (TTP_min_)) were assessed and compared with neuropathological findings and contrast-enhanced MRI including Dice similarity coefficients for spatial overlap between PET and MR signals.

**Results:**

A total of 147 patients were included (79 astrocytomas, 68 oligodendrogliomas). Contrast enhancement was present in 44/147 tumours (29.9%), most frequently in astrocytoma WHO grade 4 (8/9, 88.9%). [¹⁸F]FET PET was positive in 62/68 oligodendrogliomas (91.2%) and 35/79 astrocytomas (44.3%), with significantly higher uptake intensities in oligodendrogliomas (median TBR_max_ 2.43 vs. 1.55; *p* < 0.001) and increasing values with WHO grade. PET–CE spatial overlap was generally low (median Dice 0.26 (range: 0.01–0.90)), whereas PET–FLAIR overlap was higher (median Dice 0.55 (range: 0.05–0.99)) and showed significant differences between histologies and grades. Dynamic [¹⁸F]FET PET data were available in 91/147 tumours (61.9%) and demonstrated predominantly increasing kinetics (61.5%). In the TTP_min_ analysis, WHO grade 4 astrocytomas clustered in the early 12.5-min group, whereas WHO grade 2–3 gliomas showed later peaks (17.5–35 min).

**Conclusion:**

[^18^F]FET PET uptake intensity correlates positively with the CNS WHO grade in *IDH*-mutant gliomas, but with marked overlap between tumour grades, and provides complementary information to contrast-enhanced MRI. [^18^F]FET PET may therefore add another layer of information on tumour characteristics and should be further investigated as a complementary biomarker.

## Introduction

Mutations in the isocitrate dehydrogenase 1 and 2 (*IDH1*/*2*) genes are commonly found in diffuse gliomas previously classified as lower-grade gliomas. These mutations lead to the production of 2-hydroxyglutarate, a metabolite that disrupts physiological cellular processes, including DNA methylation and gene regulation. Due to their distinct molecular profiles, *IDH*-mutant gliomas are now classified as separate entities in the updated WHO Classification of Central Nervous System Tumours 2021 [1]. Gliomas harboring *IDH* mutations along with a 1p/19q codeletion are categorized as oligodendrogliomas, whereas those lacking this codeletion are classified as astrocytomas. Both tumour entities exhibit diffuse infiltration into adjacent brain tissue and can progress toward more aggressive forms, often accompanied by contrast enhancement (CE) on magnetic resonance imaging (MRI) [2–4].

*IDH*-mutant gliomas are graded as CNS WHO grade 2 to 4 tumours based on histological criteria such as anaplasia, necrosis, and microvascular proliferation [5]. Additionally, molecular alterations such as homozygous deletion of the CDKN2A/CDKN2B genes are increasingly considered for tumour grading. Tumour grade correlates strongly with prognosis and guides postoperative treatment decisions alongside other prognostic factors such as patient age, extent of tumour resection, and neurological status [6]. While a watch-and-wait strategy may be adopted in low-risk scenarios, tumours at higher risk of progression typically require more aggressive interventions, such as combined radio-chemotherapy with temozolomide or the PCV regimen (procarbazine, lomustine/CCNU, vincristine) [7–9].

However, these standard treatments are associated with significant short- and long-term toxicities, which pose particular concerns for younger patients likely to experience these adverse effects over a long lifetime. Consequently, innovative and better-tolerated therapeutic strategies are urgently needed. Recently, the phase 3 INDIGO trial demonstrated prolonged progression-free survival in patients with residual or recurrent WHO grade 2 *IDH*-mutant gliomas who had undergone no previous treatment other than surgery and received vorasidenib, a selective IDH inhibitor, compared to placebo. Interestingly, this trial included only patients with non-enhancing tumours, as earlier-phase studies indicated poorer responses among patients with contrast-enhancing gliomas [3, 7, 10].

Importantly, CE in MRI is not specific to neoplastic processes and can also result from inflammation, ischemia, or reactive tissue changes. Thus, alternative imaging methods are required to more accurately identify tumour activity. In this retrospective single-center study, we aimed to investigate the correlation between MRI characteristics and [^18^F]FET uptake in newly diagnosed *IDH*-mutant gliomas. Our goal is to provide foundational data for future studies aimed at establishing predictive imaging biomarkers for patient selection and monitoring treatment response to IDH inhibitors such as vorasidenib [11, 12].

## Materials and methods

### Patients

This retrospective study included patients with histologically verified *IDH*-mutant gliomas of CNS WHO grades 2 to 4 according to the 2021 WHO classification who were diagnosed between 2012 and 2024 at the LMU University Hospital of the Ludwig Maximilians University (LMU) Munich, Germany and underwent routine [^18^F]FET PET/CT imaging prior to any therapeutic intervention such as surgical resection, radiotherapy, or chemotherapy. All patients underwent stereotactic biopsy or resection at most two weeks after the initial [^18^F]FET PET scan. The study received approval from the ethical review board of LMU (approval no.17–656, 604 − 16) and was conducted in accordance with the Declaration of Helsinki. All patients provided written informed consent prior to each [^18^F]FET PET/CT scan which was performed as part of clinical routine.

### MRI

Patients underwent whole-brain MRI scans (1.5T or 3 T) including transversal T2-weigthed imaging (slice thickness 2–3 mm), 3D T2-FLAIR (fluid-attenuated inversion recovery; slice thickness 1 mm), and 3D T1-weighted imaging (slice thickness 1 mm) both before and after administration of a gadolinium-based contrast agent. CE was visually assessed, and complex cases were additionally reviewed by board-certified neuroradiologists with 6 and 12 years of experience in diagnostic neuroradiology, evaluating the presence or absence of enhancement and the heterogeneity of its distribution within the tumour volume.

### PET acquisition and data evaluation

[^18^F]FET PET/CT scans were acquired using a Biograph-64 PET/CT scanner (Siemens Healthineers, Erlangen, Germany) or an ECAT EXACT HR+ (Siemens Healthineers) at the LMU University Hospital, adhering to standard protocols [13]. Summation images from 20 to 40 min post injection were analyzed on a Hermes workstation (Hermes Medical Solutions) following established methodologies [14]. First, the mean background activity in healthy contralateral hemisphere was determined [15]. Subsequently, the PET volume was determined semiautomatically by multiplying the mean background activity by a factor of 1.6 and using the resulting SUV as lower threshold for tumour segmentation. Within the defined PET volume, both the maximum and mean tumour-to-background ratios (TBR_max_/TBR_mean_) were calculated by dividing the maximum and mean standardized uptake values (SUV_max_ and SUV_mean_), respectively, by the mean background activity. Tumours with a TBR_max_ value ≥ 1.6 were classified as [^18^F]FET-positive, in accordance with PET-RANO 1.0. To assess inter- and intra-observer reproducibility for [¹⁸F]FET positivity, two nuclear medicine physicians with different levels of experience independently evaluated all PET scans. To evaluate intra-observer reproducibility, the less experienced reader repeated the assessment.

With regard to registration accuracy and segmentation consistency, all datasets were automatically co-registered in the Hermes Hybrid Viewer (Hermes Medical Solutions) using rigid PET–MRI alignment. Each co-registration was then visually verified by cross-checking anatomical landmarks—such as skull contours, nasal structures, orbital anatomy, and the confluence of sinuses—to ensure accurate spatial matching. In cases where the automatic co-registration was imperfect, manual fine-adjustments were performed to achieve optimal alignment.

Additionally, the spatial overlap between PET volume and MRI abnormalities was quantified using the Dice coefficient. Dice coefficient values were computed only when a measurable lesion could be segmented on both PET and MRI; cases without visible FET uptake or without a segmentable MRI lesion (absence of FLAIR hyperintensity or contrast enhancement) were not included in the Dice analysis. PET-volumes were segmented on the Hermes Hybrid Viewer (Hermes Medical Solutions) using a fixed threshold of TBR ≥ 1.6. Corresponding MRI lesions (FLAIR hyperintensity and contrast-enhancing regions) were manually segmented on the same Hermes platform to ensure precise spatial correspondence. For each evaluable tumour, the Dice coefficient was calculated as:

$$\:DICE=\frac{2\times\:\mid\:{V}_{PET}\cap\:{V}_{MRI}\mid\:}{\mid\:{V}_{PET}\mid\:+\mid\:{V}_{MRI}\mid\:}$$

where $\:{V}_{PET}$​ refers to the PET volume, $\:{V}_{MRI}$ to the respective MRI lesion volume, and $\:\mid\:{V}_{PET}\cap\:{V}_{MRI}\mid\:$ to their volumetric intersection. Dice values were obtained separately for PET–FLAIR and PET–contrast enhancement comparisons and analysed by histological subtype and WHO grade.

### Dynamic [¹⁸F]FET PET analysis

Dynamic [¹⁸F]FET PET data were evaluated when a dynamic acquisition (0–40 min post-injection) was performed.All datasets were reconstructed identically and analyzed on a Hermes workstation (Hermes Medical Solutions). Regions of interest were defined using a 90% isocontour threshold on the summation image (10–30 min post-injection) and applied uniformly to the full dynamic dataset to extract time–activity curves. Thus, identical frame segmentation and time bins were used for all patients, ensuring consistent kinetic analysis and reproducibility across the cohort [16]. Tumours were categorized as showing increasing time–activity curves when standardized uptake values (SUVs) continuously increased or reached a peak followed by a plateau in the subsequent frames throughout the dynamic acquisition. Decreasing time–activity curves were defined by an initial peak followed by a constant decline in SUV values, occurring either homogeneously throughout the tumour or heterogeneously in at least two adjacent slices [17]. Stable uptake patterns were defined when no clear increasing or decreasing trend was observed, with SUV fluctuations remaining within ± 10% of the maximal peak value over time. For each patient, minimal time-to-peak (TTP_min_) was evaluated across all tumour slices, and the shortest TTP observed in at least two consecutive slices was defined as the minimal time-to-peak (TTP_min_), in accordance with previously published methodology [16, 18].

### Histological verification, tumour grading, and molecular genetic analysis

Histological and molecular genetic analyses on biopsied/resected tumour tissue were performed at the Institute of Neuropathology, LMU Munich, and tumours were (re)classified according to the 2021 WHO classification by a board-certified neuropathologist [1].

### Statistical analysis

Metric data are reported as median (range), and categorical variables as absolute numbers and percentages. Group differences in categorical variables—including PET kinetic patterns (increasing, stable, decreasing), availability of TTP_min_ values, and Dice-overlap categories—were analysed using the Chi-square test, with χ² statistics and corresponding p-values presented for all comparisons. Differences in metric variables such as Dice coefficients and tumour volumes were assessed using the Mann–Whitney U test. Inter-reader and intra-reader reproducibility for the assessment of PET positivity (TBR_max_ ≥ 1.6) was evaluated using Cohen’s κ (kappa). Statistical significance was defined as a two-tailed p-value < 0.05. Given the hypothesis-generating nature of this study, no correction for multiple testing was applied [19]. Statistical analyses were performed with SPSS (Version 29.0.0.0, SPSS Inc., Chicago, IL).

## Results

### Patients’ characteristics

Overall, 147 patients (61/147 [41.5%] females, 86/147 [58.5%] males; median age 45 years, range 25–89) with newly diagnosed *IDH*-mutant CNS WHO grade 2–4 glioma were included in this study (Table 1). Neuropathological analysis identified 79/147 (53.4%) astrocytomas and 68/147 (46.3%) oligodendrogliomas. In total, 101/147 (68.7%) were classified as CNS WHO grade 2, 37/147 (25.2%) as CNS WHO grade 3, and 9/147 (6.1%) as CNS WHO grade 4.

**Table 1:** Patient demographics and histological classification

Characteristics	Number of patients (*n* = 147)
Sex	
- Female	61 (41.5%)
- Male	86 (58.5%)
Median age (years)	45 (range: 25–89)
Histological subtype	
*Astrocytoma*,* IDH-mutant*	79 (53.4%)
- CNS WHO grade 2	49 *(62.0%)*
- CNS WHO grade 3	21 *(26.6%)*
- CNS WHO grade 4	9 *(11.4%)*
*Oligodendroglioma*,* IDH-mutant*,* 1p/19q-codeleted*	68 (46.3%)
- CNS WHO grade 2	52 *(76.5%)*
- CNS WHO grade 3	16 *(23.5%)*

### Imaging characteristics according to tumour subtype and WHO grade

**Fig. 1:**
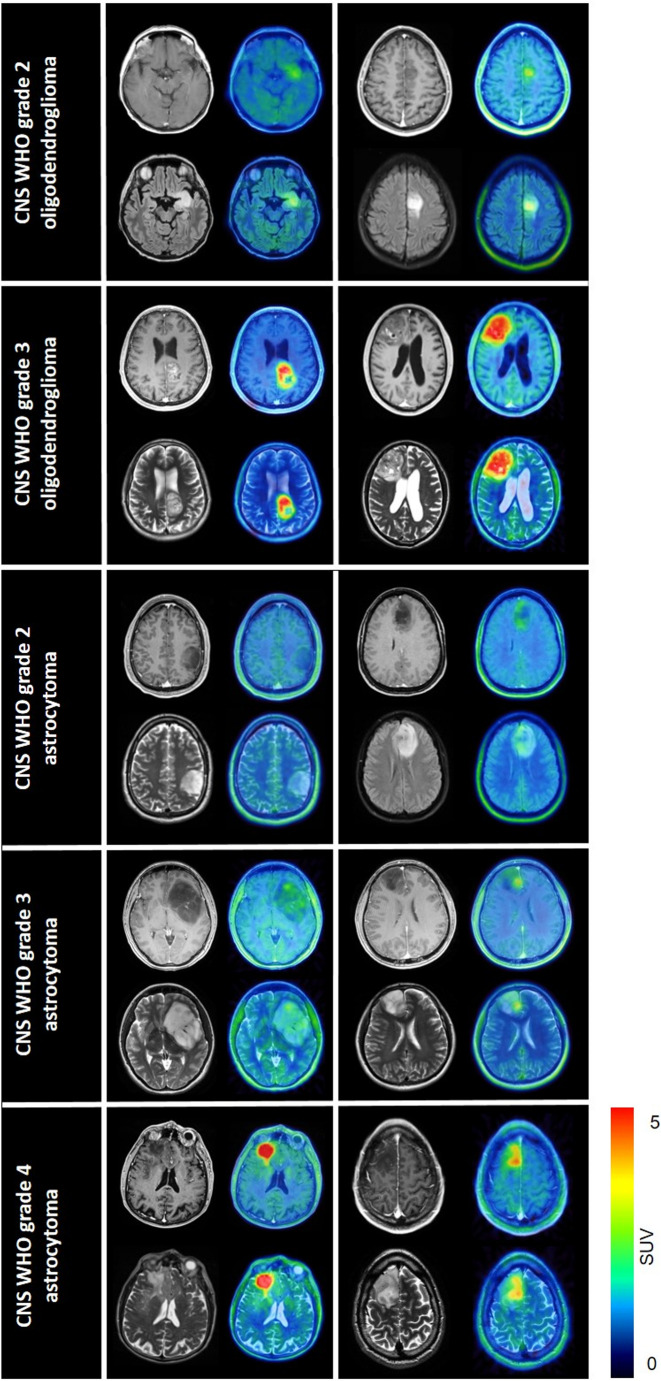
Examples for MRI/[^18^F]FET PET imaging in subgroups of *IDH*-mutant glioma (two examples per entity). Upper panels: contrast-enhanced T1; lower panels: T2/FLAIR; left panels: MRI; right panels: fused MRI/[^18^F]FET PET

**Fig. 2:**
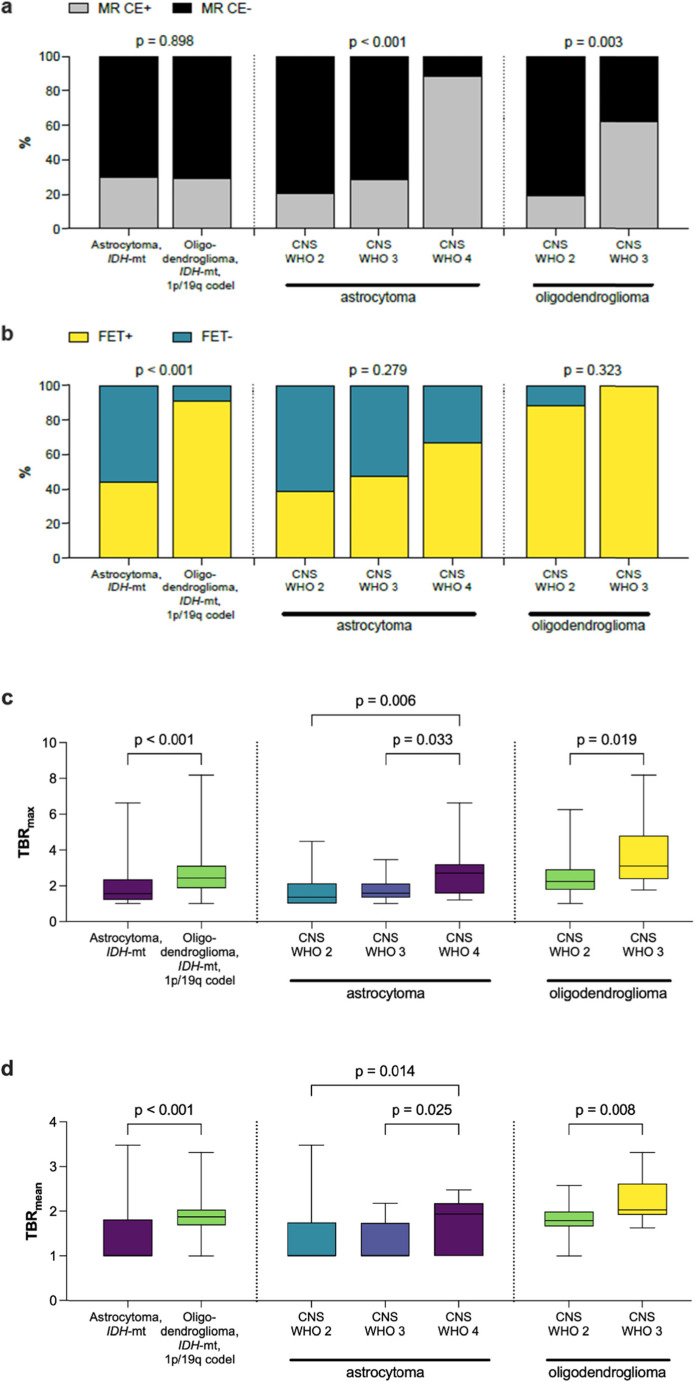
Quantitative and semiquantitative parameters of MR and [^18^F]FET PET imaging in molecular subgroups of *IDH-*mutant gliomas. (**a**) Contrast enhancement (CE+); (**b**) FET positivity; (**c**) TBR_max_ and (**d**) TBR_mean_. *P*-values as determined by (**a**/**b**) Chi-square or Fisher’s exact tests and (**c**/**d**) pairwise Mann-Whitney-U test as appropriate

Illustrative examples for MRI and PET images are shown in Fig. 1. On MRI, 44/147 (29.9%) cases showed contrast enhancement (Fig. 2a). This included 24/79 astrocytomas (30.4%) and 20/68 oligodendrogliomas (29.4%; *p* = 0.898). Among CNS WHO grade 2 astrocytomas, 10/49 (20.4%) were contrast-enhancing, compared to 6/21 (28.6%) of CNS WHO grade 3 and 8/9 (88.9%) of CNS WHO grade 4 (*p* < 0.001). Among CNS WHO grade 2 oligodendrogliomas, 10/52 (19.2%) showed contrast enhancement, compared to 10/16 (62.5%) CNS WHO grade 3 (*p* = 0.003).

[^18^F]FET PET analyses demonstrated distinct imaging characteristics between astrocytomas and oligodendrogliomas. Overall, 97/147 (66.0%) cases were [^18^F]FET-positive, with a significant difference in PET-positivity rates between oligodendrogliomas (62/68, 91.2%) and astrocytomas (35/79, 44.3%; *p* < 0.001; Fig. 2b). Reproducibility of [¹⁸F]FET positivity classification was high (inter-observer κ = 0.87; intra-observer κ = 0.86). In line, TBR_max_ was higher in oligodendrogliomas (2.43; 1.0–8.18) compared to astrocytomas (1.55; 1.0–6.63; *p* < 0.001; Fig. 2c). Similarly, TBR_mean_ values were higher in oligodendrogliomas (1.88; 1.0–3.32) compared to astrocytomas (1.46; 1.0–3.48; *p* < 0.001, Fig. 2d).

[^18^F]FET uptake showed a positive correlation with WHO grade, albeit with notable overlap of TBR_max_ and TBR_mean_ values across WHO grades (Fig. 2c**/**d). There was no difference in the TBR_max_ between CNS WHO grade 2 (1.35; 1.0–4.48) and CNS WHO grade 3 astrocytomas (1.58; 1.0–3.45.0.45, *p* = 0.548). However, TBR_max_ was significantly higher in CNS WHO grade 4 astrocytoma (2.70; 1.0–6.63.0.63) compared to CNS WHO grade 2 (*p* = 0.006) and CNS WHO grade 3 (*p* = 0.033). In oligodendrogliomas, a significant difference in TBR_max_ values was noted between CNS WHO grades 2 and 3 (2.24; 1.0–6.25.0.25 vs. 3.10; 1.76–8.18, *p* = 0.019).

### Dynamic [¹⁸F]FET PET kinetics

Dynamic [¹⁸F]FET PET data were evaluated in 91/97 PET-positive tumours (93.8%), whereas the remaining 6 cases lackedkinetic information due to technical issues during dynamic acquisition or due to static acquisition. Most cases presented with increasing time activity curves (56/91; 61.5%), followed by stable (22/91; 24.2%) and decreasing curves (13/91; 14.3%). Inter-reader agreement for kinetic pattern classification was κ = 0.93 and Intra-reader agreement was κ = 0.98.

Stratified by WHO grade, WHO grade 2 tumours most frequently showed increasing time activity curves (41/59; 69.5%), with fewer stable (13/59; 22.0%) and decreasing patterns (5/59; 8.5%). WHO grade 3 tumours (*n* = 26) demonstrated a more even distribution (increasing 50.0%, stable 34.6%, decreasing 15.4%). In contrast, WHO grade 4 astrocytomas (*n* = 6) were characterised by a predominance of decreasing kinetics (4/6; 66.7%), with the remaining cases showing increasing uptake (33.3%) and no stable curves. Kinetic patterns differed significantly across WHO grades (χ² = 17.6, *p* = 0.001). Within the subgroups, dynamic information was available in 33/79 astrocytomas and 58/68 oligodendrogliomas. Astrocytomas showed predominantly increasing kinetics (24/33; 72.7%), with stable (15.2%) and decreasing patterns (12.1%) less common. Oligodendrogliomas exhibited a more balanced distribution, with increasing curves in 32/58 (55.2%), stable in 17/58 (29.3%), and decreasing in 9/58 (15.5%). The distribution of kinetic patterns did not differ significantly between astrocytomas and oligodendrogliomas (χ² = 3.0, *p* = 0.23).

Minimal time-to-peak (TTP_min_) analysis in astrocytomas and oligodendrogliomas showed identical overall median TTP_min_ values (35.0 min each), and no significant difference between groups was observed (Mann–Whitney U = 931.5, *p* = 0.89). Among astrocytomas, WHO grade 2 and 3 tumours exhibited uniformly late peak times (median 35.0 min), whereas WHO grade 4 astrocytomas clustered distinctly in the early-peak range (median 12.5 min). This distribution differed significantly across astrocytoma grades (Kruskal–Wallis H = 22.4, *p* < 0.001), driven by markedly earlier peaks in WHO grade 4 tumours (WHO grade 4 vs. WHO grade 2: *p* < 0.001; WHO grade 4 vs. WHO grade 3: *p* = 0.002), while WHO grades 2 and 3 did not differ significantly (*p* = 0.91). Among oligodendrogliomas, WHO grade 2 tumours demonstrated uniformly late peak times (median 35.0 min), whereas WHO grade 3 lesions showed slightly earlier peaks (median 25.0 min; Mann–Whitney U = 244.0, *p* = 0.014).

### [^18^F]FET PET uptake patterns and correlation with MRI

**Fig. 3:**
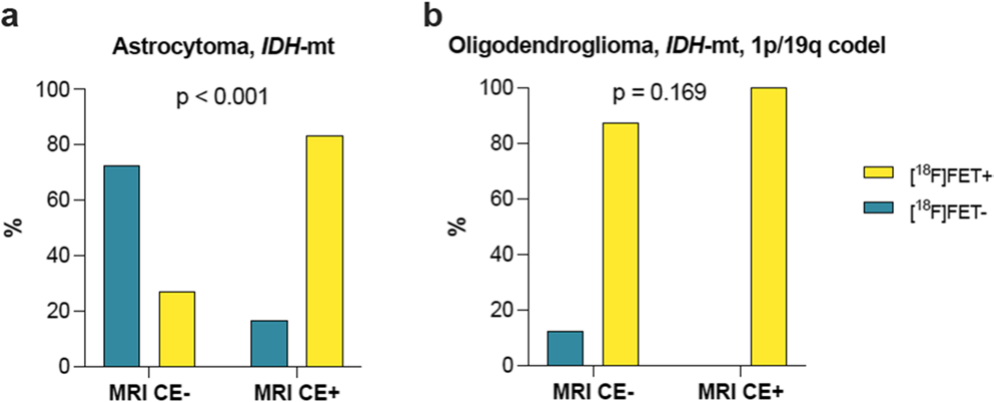
Association of MRI with [^18^F]FET PET imaging characteristics. Contrast enhancement (CE+/CE-) with FET positivity in (**a**) astrocytoma and (**b**) oligodendroglioma; *P*-values as determined by Chi-square or Fisher’s exact tests as appropriate

Astrocytomas with contrast enhancement (CE+) demonstrated substantially higher occurrence of [^18^F]FET uptake (20/24, 83.3%) compared to astrocytomas without contrast enhancement (CE-) (15/55, 27.3%; *p* < 0.001, Fig. 3a). All CE + oligodendrogliomas (20/20) and most CE- oligodendrogliomas (42/48, 87.5%) were also [^18^F]FET-positive (*p* = 0.169, Fig. 3b).

In the subgroup of contrast-enhancing tumours, [¹⁸F]FET uptake frequently extended beyond the enhancing region on MRI. Quantitatively, Contrast-enhancing (CE)–PET dice coefficient in 38 lesions and was generally low across *IDH*-mutant glioma as illustraded in Table 2. Dice coefficients were markedly lower in WHO grade 2–3 gliomas, with similar values in astrocytomas (WHO grade 2: 0.14 (0.06–0.78); WHO grade 3: 0.21 (0.13–0.35)) and oligodendrogliomas (WHO grade 2: 0.08 (0.07–0.83); WHO grade 3: 0.20 (0.01–0.33)). In contrast, WHO grade 4 astrocytomas demonstrated substantially higher CE–PET overlap (0.61 (0.08–0.90)). A Kruskal–Wallis test showed significant differences across groups (H = 14.82, *p* = 0.005), driven exclusively by the marked increase in dice coefficient values in WHO grade 4 astrocytomas, which were significantly higher than all WHO grade 2–3 tumours (all post-hoc p-values < 0.04). No significant differences were found among the WHO grade 2–3 subgroups.

**Table 2:** Dice coefficients for PET–Contrast-enhancing MRI Spatial overlap across histology and WHO grade

Histology	Number of patients	Dice (median ± range)
*Astrocytoma*,* IDH-mutant*	18	0.21 (0.06–0.90)
- CNS WHO grade 2	7	0.14 (0.06–0.78)
- CNS WHO grade 3	4	0.21 (0.13–0.35)
- CNS WHO grade 4	7	0.61 (0.08–0.90)
*Oligodendroglioma*,* IDH-mutant*,* 1p/19q-codeleted*	20	0.17 (0.01–0.83)
- CNS WHO grade 2	10	0.08 (0.07–0.83)
- CNS WHO grade 3	10	0.20 (0.01–0.33)

In the 87 PET-measurable lesions, PET–FLAIR dice coefficient differed substantially across histological subtypes and WHO grades. As illustrated in Table 3 among astrocytomas, dice coefficient values showed a significant overall difference between WHO grades (Kruskal–Wallis H = 9.13, *p* = 0.010). WHO grade 4 astrocytomas demonstrated markedly higher Dice coefficients (0.71 (0.55–0.96)) compared with WHO grade 2 (0.33 (0.07–0.91), *p* = 0.013) and WHO grade 3 tumours (0.31 (0.11–0.49), *p* = 0.001), whereas WHO grade 2 and grade 3 astrocytomas did not differ from each other (*p* = 0.664). In oligodendrogliomas, PET–FLAIR concordance also increased with malignancy: WHO grade 3 tumours showed significantly higher dice coefficient values 0.77 (0.05–0.99)) compared with WHO grade 2 (0.63 (0.05–0.95), *p* = 0.021). When comparing histologies, oligodendrogliomas exhibited significantly higher morpho-metabolic concordance than astrocytomas overall (*p* = 0.005).

**Table 3:** Dice coefficients for PET–FLAIR Spatial overlap across histology and WHO grade

Histology	Number of patients	Dice (median ± range)
*Astrocytoma*,* IDH-mutant*	29	0.44 (0.07–0.96)
- CNS WHO grade 2	16	0.33 (0.07–0.91)
- CNS WHO grade 3	7	0.31 (0.11–0.49)
- CNS WHO grade 4	6	0.71 (0.55–0.96)
*Oligodendroglioma*,* IDH-mutant*,* 1p/19q-codeleted*	58	0.72 (0.05–0.99)
- CNS WHO grade 2	42	0.63 (0.05–0.95)
- CNS WHO grade 3	16	0.77 (0.05–0.99)

## Discussion

In this retrospective study, we investigated the correlation between PET characteristics and MRI findings in newly diagnosed *IDH*-mutant gliomas. While contrast enhancement occurred similarly across astrocytomas and oligodendrogliomas, clear differences emerged concerning tumour grade. Contrast enhancement generally indicates blood-brain barrier disruption, typically associated with higher tumour malignancy [20]. Nonetheless, our findings support earlier observations that non-enhancing tumours can also exhibit higher histological grades, complicating clinical assessment based solely on MRI [21]. For instance, a substantial percentage in CNS WHO grade 2 gliomas exhibited contrast enhancement in our cohort, corroborating previous data [22, 23]. This is of particular interest given the advent of IDH inhibitors such as vorasidenib in *IDH-*mutant CNS WHO grade 2 gliomas, showing prolonged progression-free survival compared to observation and overall higher response rates in non-enhancing gliomas [2, 3]. These findings underscore the need for additional biomarkers beyond conventional MRI characteristics to better guide therapeutic decisions. Given the diagnostic and prognostic ambiguities inherent in differentiating CNS WHO grade 2 from grade 3 gliomas, enhanced imaging biomarkers could provide critical assistance in clinical decision-making, particularly in contexts where inter-observer variability remains high [24]. In this regard, epigenetic classification has resulted in continuous grading frameworks correlating with outcome and enriched cellular pathways in these tumours [25, 26].

Consistent with previous literature, our results demonstrate a correlation between increasing tumour grade and elevated [^18^F]FET PET uptake, notably more pronounced in oligodendrogliomas compared to astrocytomas [27, 28]. In our cohort, only < 10% of oligodendrogliomas lacked [^18^F]FET uptake, whereas this was the case in > 50% of astrocytomas, highlighting the importance of histological workup. Recent findings further support that amino acid PET parameters, particularly TBR_max_, TBR_mean_, and PET volume, are prognostically relevant in *IDH*-mutant gliomas, with stronger associations observed in astrocytomas [29]. Higher TBR_max_, TBR_mean_, and PET volume were associated with shorter time to next intervention and worse overall survival. Still, the underlying mechanisms behind differential uptakes of astrocytomas and oligodendrogliomas characteristics remain poorly elucidated, and the higher metabolic activity in amino acid PET appears counterintuitive given the overall slower growth and comparably better prognosis of oligodendrogliomas. Amino acid PET uptake was reported to correlate with the expression of L-amino acid transporters, although comparative studies in contemporary cohorts of gliomas classified by the current WHO classification are lacking [30–32]. Other hypotheses extend to a higher vascularization in oligodendrogliomas compared to astrocytomas leading to more frequent contrast enhancement at similar grades [33, 34]. Moreover, L-amino acid transporters were found to be frequently expressed on endothelial cells, potentially explaining higher uptake in oligodendrogliomas [30]. Systematic investigations correlating L-amino acid transporter expression as well as genetic and epigenetic profiles with PET uptake characteristics are needed to elucidate the underlying biology behind differential uptake between glioma subtypes and its clinical relevance.

Moreover, dynamic [¹⁸F]FET PET revealed biologically meaningful differences in uptake behaviour across *IDH*-mutant glioma subtypes. Lower-grade tumours (WHO 2–3) predominantly showed increasing or stable uptake curves, consistent with delayed tracer accumulation, whereas WHO grade 4 astrocytomas shifted toward decreasing kinetics with early peak times, reflecting more aggressive metabolic behaviour. Oligodendrogliomas exhibited a broader kinetic spectrum but largely followed the same pattern, with slower, later peaks in lower-grade disease and earlier peaks in higher-grade lesions. Together, these findings support the notion that dynamic [¹⁸F]FET PET provides additional biological discrimination beyond static TBR measures, particularly in separating lower grade *IDH*-mutant gliomas from WHO grade 4 astrocytomas.

In our cohort, FLAIR hyperintensities in MRI frequently exceeded volumes showing [^18^F]FET uptake, whereas CE + volumes were commonly inferior to PET volume in both astrocytomas and oligodendrogliomas. Indeed, previous studies have shown mismatches between CE + volumes, [^18^F]FET uptake and areas of high malignancy as evaluated by biopsies [35, 36].

The complementary information provided by PET is not only relevant for biopsy guidance but also for local therapeutic interventions. Maximal safe resection remains the mainstay of surgical treatment with an increasing body of evidence supporting supramaximal resection beyond radiological tumour borders while maintaining eloquent areas [37]. Resection of all PET volume showed improved oncological outcomes in high-grade gliomas and glioblastoma [38–41]. Moreover, PET may provide additional information for the definition of radiotherapeutic target volumes as increasingly reflected in guidelines and consensus statements [42, 43]. In particular, PET allows to identify areas of high metabolic activity beyond alterations in MRI and to differentiate edema from active tumour tissue in T2/FLAIR hyperintensities. However, due to the retrospective nature of our study, no spatially matched tissue samples from different imaging-defined regions were available to validate whether FET-positive areas indeed represent tumour infiltration or whether T2/FLAIR abnormalities exceeding [¹⁸F]FET uptake reflect edema or tumour with low metabolic activity. Furthermore, studies evaluating the influence of amino acid PET on surgical and radiotherapy planning are needed to corroborate an impact on oncological and functional outcomes.

We additionally incorporated a quantitative assessment of PET–MRI spatial relationships by calculating Dice coefficients for both PET–FLAIR and PET–contrast-enhancing lesion overlap. These data demonstrated that spatial concordance between metabolic and morphological tumour extent varies markedly across *IDH*-mutant glioma subtypes. PET–FLAIR overlap was highest in WHO grade 4 astrocytomas and oligodendroglioma WHO grade 3, whereas lower-grade astrocytomas showed substantially more discordant patterns, suggesting a more heterogeneous and infiltrative tumour architecture. PET–CE Dice values were consistently low across all subgroups; however, astrocytoma WHO grade 4 exhibited noticeably higher PET–CE overlap compared with lower-grade tumours, underscoring the stronger coupling between metabolic activity and blood–brain barrier breakdown in higher malignancy. Although the number of enhancing tumours—particularly in grades 2–3—was limited, these analyses reinforce the complementary diagnostic value of amino-acid PET, especially when MRI fails to capture metabolically active tumour regions.

Nonetheless, these findings must be interpreted in light of the limited number of PET-measurable tumours, particularly within the astrocytoma WHO grade 3 and 4 subgroups. Larger, multicentric cohorts will be required to confirm these patterns and establish robust reference values for clinical use. Low Dice values highlight metabolically active foci within diffusely infiltrative tumour components that may be missed by MRI alone. Together, these results underscore the complementary nature of amino acid PET and MRI. Although our dataset does not allow assessment of treatment response or prognostic value, the presence of focal high-uptake regions invisible on MRI suggests that amino acid PET may identify biologically active tumour compartments. This concept is supported by recent studies demonstrating prognostic associations and potential prognostic value of amino acid PET in *IDH*-mutant gliomas [29]. Integrating prospective trials will be essential to determine its role in stratifying patients for IDH-targeted therapy. Although current EANO and NCCN guidelines primarily rely on contrast-enhanced MRI, our findings and recent data by Mair et al. suggest that [¹⁸F]FET PET can add a valuable functional–metabolic layer to the diagnostic pathway. PET-derived parameters such as PET volume and TBR_max_ may offer superior risk stratification compared to neuropathological grading alone, supporting the hypothesis that amino acid PET may more accurately reflect the biological behaviour of *IDH*-mutant gliomas than tissue-based sampling.

Although the well-characterized, homogenous cohort classified according to the WHO 2021 classification is a strength of the study, there are important limitations such as the retrospective design, and small sample sizes in certain subgroups. Furthermore, different PET and MRI scanners were used over the study period, reflecting real-world clinical conditions. PET scans have been performed using different scanners, limiting the homogeneity and comparability of scans. This may have introduced minor variability in SUV quantification and tumor delineation despite the use of standardized imaging procedures. Moreover, in future studies, it would be interesting to correlate PET findings to advanced MRI techniques such as diffusion-weighted imaging (DWI) or perfusion-weighted imaging (PWI), which could serve as surrogate markers for cellularity, proliferation, or neuroangiogenesis. To minimize these differences, standardized protocols were used for image acquisition and analysis.

Given the exploratory nature of our study, the small subgroup sizes (e.g., WHO grade 4, *n* = 9) limit statistical power, and future multicentric studies with larger cohorts are needed to validate these findings. While exploratory correlations between [¹⁸F]FET PET parameters and molecular features such as CDKN2A/B homozygous deletion or methylation-based prognostic profiles would be of high interest, these analyses are planned within upcoming multicentric projects given the low prevelance of CDKN2A/B homozygous deletion [44]. As most previous studies have been based on earlier WHO classifications and included *IDH*-wildtype gliomas, our current work aimed to provide a comprehensive descriptive analysis of [¹⁸F]FET PET characteristics in a purely *IDH*-mutant glioma cohort. The correlation between [¹⁸F]FET PET characteristics, tumor grade and presence of contrast enhancement is of particular interest in the light of the INDIGO trial and use of Vorasidenib, as tumor grade and contrast enhancement have been described as biomarkers used for patient selection. Lastly, precise spatial validation of tumour extent was not feasible in this retrospective cohort. Without spatially matched histopathological sampling, it remains impossible to determine which modality—MRI or [¹⁸F]FET PET—more accurately reflects true tumour infiltration. In conclusion, we could show that [^18^F]FET PET correlates with tumour grade and provides complementary information to contrast-enhanced MRI in *IDH*-mutant glioma. Future prospective studies should explore genetic, epigenetic, and molecular correlates of PET uptake, aiming to validate its role as a predictive biomarker for treatment selection and monitoring in the evolving landscape of *IDH*-mutant glioma management.

## Data Availability

Data reported in this article can be shared in compliance with current data protection regulations by the European Union. Data sharing requires a current and positive vote by the requestor´s competent ethics committee. All proposals should be directed to the corresponding author and data requestors will need to sign a data access agreement with the LMU Univesity Hospital.

## References

[1] Louis DN, et al. The 2021 WHO classification of tumors of the central nervous system: a summary. Neuro Oncol. 2021;23(8):1231–51. 34185076 10.1093/neuonc/noab106 PMC8328013

[2] Mellinghoff IK, et al. Vorasidenib in IDH1- or IDH2-Mutant Low-Grade glioma. N Engl J Med. 2023;389(7):589–601. 37272516 10.1056/NEJMoa2304194 PMC11445763

[3] Mellinghoff IK, et al. Vorasidenib, a dual inhibitor of mutant IDH1/2, in recurrent or progressive Glioma; results of a First-in-Human phase I trial. Clin Cancer Res. 2021;27(16):4491–9. 34078652 10.1158/1078-0432.CCR-21-0611 PMC8364866

[4] Miller JJ, et al. Isocitrate dehydrogenase (IDH) mutant gliomas: A society for Neuro-Oncology (SNO) consensus review on diagnosis, management, and future directions. Neuro Oncol. 2023;25(1):4–25. 36239925 10.1093/neuonc/noac207 PMC9825337

[5] Reuss DE. Updates on the WHO diagnosis of IDH-mutant glioma. J Neurooncol. 2023;162(3):461–9. 36717507 10.1007/s11060-023-04250-5 PMC10227121

[6] Mair MJ, et al. A basic review on systemic treatment options in WHO grade II-III gliomas. Cancer Treat Rev. 2021. 10.1016/j.ctrv.2020.102124. 33227622 10.1016/j.ctrv.2020.102124

[7] Lin MD, et al. Treatment of IDH-mutant glioma in the INDIGO era. NPJ Precis Oncol. 2024;8(1):149. 39025958 10.1038/s41698-024-00646-2 PMC11258219

[8] Reuss DE, et al. IDH mutant diffuse and anaplastic Astrocytomas have similar age at presentation and little difference in survival: a grading problem for WHO. Acta Neuropathol. 2015;129(6):867–73. 25962792 10.1007/s00401-015-1438-8 PMC4500039

[9] Carstam L, et al. WHO grade loses its prognostic value in molecularly defined diffuse Lower-Grade gliomas. Front Oncol. 2021;11:803975. 35083156 10.3389/fonc.2021.803975 PMC8785215

[10] Johannessen TA, et al. Rapid conversion of mutant IDH1 from driver to passenger in a model of human gliomagenesis. Mol Cancer Res. 2016;14(10):976–83. 27430238 10.1158/1541-7786.MCR-16-0141 PMC5065766

[11] Wollring MM, et al. Clinical applications and prospects of PET imaging in patients with IDH-mutant gliomas. J Neurooncol. 2023;162(3):481–8. 36577872 10.1007/s11060-022-04218-x PMC10227162

[12] Langen KJ, et al. Advances in neuro-oncology imaging. Nat Rev Neurol. 2017;13(5):279–89. 28387340 10.1038/nrneurol.2017.44

[13] Law I, et al. Joint EANM/EANO/RANO practice guidelines/SNMMI procedure standards for imaging of gliomas using PET with radiolabelled amino acids and [(18)F]FDG: version 1.0. Eur J Nucl Med Mol Imaging. 2019;46(3):540–57. 30519867 10.1007/s00259-018-4207-9 PMC6351513

[14] Jansen NL, et al. *MRI-suspected low-grade glioma: is there a need to perform dynamic FET PET?* Eur J Nucl Med Mol Imaging. 2012;39(6):1021–9. 22491781 10.1007/s00259-012-2109-9

[15] Unterrainer M, et al. Towards standardization of 18F-FET PET imaging: do we need a consistent method of background activity assessment? EJNMMI Res. 2017;7(1):48. 28560582 10.1186/s13550-017-0295-y PMC5449315

[16] Kunz M, et al. Dynamic 18F-FET PET is a powerful imaging biomarker in gadolinium-negative gliomas. Neuro Oncol. 2019;21(2):274–84. 29893965 10.1093/neuonc/noy098 PMC6374762

[17] Jansen NL, et al. Dynamic < sup > 18 F-FET PET in newly diagnosed astrocytic Low-Grade glioma identifies High-Risk patients. J Nucl Med. 2014;55(2):p198–203. 10.2967/jnumed.113.122333 24379223

[18] Filss CP, et al. O-(2-[(18)F]fluoroethyl)-L-tyrosine PET in gliomas: influence of data processing in different centres. EJNMMI Res. 2017;7(1):64. 28815478 10.1186/s13550-017-0316-x PMC5559408

[19] Bender R, Lange S. Adjusting for multiple testing–when and how? J Clin Epidemiol. 2001;54(4):343–9. 11297884 10.1016/s0895-4356(00)00314-0

[20] Krigers A, et al. The diagnostic value of contrast enhancement on MRI in diffuse and anaplastic gliomas. Acta Neurochir (Wien). 2022;164(8):2035–40. 35018531 10.1007/s00701-021-05103-8

[21] Alkanhal H, et al. Differentiating nonenhancing grade II gliomas from grade III gliomas using diffusion tensor imaging and dynamic susceptibility contrast MRI. World Neurosurg. 2021;146:e555–64. 33152494 10.1016/j.wneu.2020.10.144

[22] Pallud J, et al. Prognostic significance of imaging contrast enhancement for WHO grade II gliomas. Neuro Oncol. 2009;11(2):176–82. 18697954 10.1215/15228517-2008-066 PMC2718989

[23] Castet F, et al. Contrast-enhancement in supratentorial low-grade gliomas: a classic prognostic factor in the molecular age. J Neurooncol. 2019;143(3):515–23. 31054099 10.1007/s11060-019-03183-2

[24] van den Bent MJ. Interobserver variation of the histopathological diagnosis in clinical trials on glioma: a clinician’s perspective. Acta Neuropathol. 2010;120(3):297–304. 20644945 10.1007/s00401-010-0725-7 PMC2910894

[25] Ghisai SA, et al. Epigenetic landscape reorganisation and reactivation of embryonic development genes are associated with malignancy in IDH-mutant Astrocytoma. Acta Neuropathol. 2024;148(1):50. 39382765 10.1007/s00401-024-02811-0 PMC11464554

[26] Shirahata M, et al. Novel, improved grading system(s) for IDH-mutant astrocytic gliomas. Acta Neuropathol. 2018;136(1):153–66. 29687258 10.1007/s00401-018-1849-4

[27] Fueger BJ, et al. Correlation of 6-18F-fluoro-L-dopa PET uptake with proliferation and tumor grade in newly diagnosed and recurrent gliomas. J Nucl Med. 2010;51(10):1532–8. 20847166 10.2967/jnumed.110.078592

[28] Nakajo K, et al. Diagnostic performance of [(11)C]methionine positron emission tomography in newly diagnosed and untreated glioma based on the revised World Health Organization 2016 classification. World Neurosurg. 2021;148:e471–81. 33444827 10.1016/j.wneu.2021.01.012

[29] Mair MJ, et al. Prognostic stratification of newly diagnosed IDH-mutant gliomas by [18F]fluoroethyltyrosine and [11 C]methionine PET - a retrospective, bicentric cohort study. Neuro Oncol. 2025. 10.1093/neuonc/noaf196 PMC12916739 40856188

[30] Okubo S, et al. Correlation of L-methyl-11 C-methionine (MET) uptake with L-type amino acid transporter 1 in human gliomas. J Neurooncol. 2010;99(2):217–25. 20091333 10.1007/s11060-010-0117-9

[31] Wiriyasermkul P, et al. Transport of 3-fluoro-L-α-methyl-tyrosine by tumor-upregulated L-type amino acid transporter 1: a cause of the tumor uptake in PET. J Nucl Med. 2012;53(8):1253–61. 22743251 10.2967/jnumed.112.103069

[32] Youland RS, et al. The role of LAT1 in (18)F-DOPA uptake in malignant gliomas. J Neurooncol. 2013;111(1):11–8. 23086431 10.1007/s11060-012-0986-1 PMC3907171

[33] Law M, et al. Glioma grading: sensitivity, specificity, and predictive values of perfusion MR imaging and proton MR spectroscopic imaging compared with conventional MR imaging. AJNR Am J Neuroradiol. 2003;24(10):1989–98. 14625221 PMC8148904

[34] Smits M. Imaging of oligodendroglioma. Br J Radiol. 2016;89(1060):20150857. 26849038 10.1259/bjr.20150857 PMC4846213

[35] Song S, et al. Simultaneous FET-PET and contrast-enhanced MRI based on hybrid PET/MR improves delineation of tumor Spatial biodistribution in gliomas: a biopsy validation study. Eur J Nucl Med Mol Imaging. 2020;47(6):1458–67. 31919633 10.1007/s00259-019-04656-2 PMC7188715

[36] Harat M, et al. Combining amino acid PET and MRI imaging increases accuracy to define malignant areas in adult glioma. Nat Commun. 2023;14(1):4572. 37516762 10.1038/s41467-023-39731-8 PMC10387066

[37] Karschnia P, et al. The oncological role of resection in newly diagnosed diffuse adult-type glioma defined by the WHO 2021 classification: a review by the RANO resect group. Lancet Oncol. 2024;25(9):e404–19. 39214112 10.1016/S1470-2045(24)00130-X

[38] Pirotte BJ, et al. Positron emission tomography-guided volumetric resection of supratentorial high-grade gliomas: a survival analysis in 66 consecutive patients. Neurosurgery. 2009;64(3):471–81. discussion 481. 19240609 10.1227/01.NEU.0000338949.94496.85

[39] Ort J, et al. (18)F-FET-PET-guided gross total resection improves overall survival in patients with WHO grade III/IV glioma: moving towards a multimodal imaging-guided resection. J Neurooncol. 2021;155(1):71–80. 34599479 10.1007/s11060-021-03844-1 PMC8545732

[40] Hirono S, et al. Oncological and functional outcomes of supratotal resection of IDH1 wild-type glioblastoma based on (11)C-methionine PET: a retrospective, single-center study. Sci Rep. 2021;11(1):14554. 34267303 10.1038/s41598-021-93986-z PMC8282858

[41] Ohmura K, et al. Resection of positive tissue on methionine-PET is associated with improved survival in glioblastomas. Brain Behav. 2023;13(12):e3291. 37846176 10.1002/brb3.3291 PMC10726771

[42] Galldiks N, et al. Contribution of PET imaging to radiotherapy planning and monitoring in glioma patients - a report of the PET/RANO group. Neuro Oncol. 2021;23(6):881–93. 33538838 10.1093/neuonc/noab013 PMC8168815

[43] Baumert BG, et al. ESTRO-EANO guideline on target delineation and radiotherapy for IDH-mutant WHO CNS grade 2 and 3 diffuse glioma. Radiother Oncol. 2025;202:110594. 39454886 10.1016/j.radonc.2024.110594

[44] Nakasu S, et al. Prognostic implication of CDKN2A/B homozygous deletion on histological grades in isocitrate dehydrogenase-mutant astrocytomas: A systematic review and meta-analysis. Neurooncol Adv. 2025;7(1):vdaf171. 40980451 10.1093/noajnl/vdaf171 PMC12448685

